# Landmark matching and B-spline implicit neural representations for diffusion-weighted imaging distortion correction

**DOI:** 10.1088/1361-6560/ae4162

**Published:** 2026-02-17

**Authors:** Yunxiang Li, Yen-Peng Liao, Yan Dai, Jie Deng, You Zhang

**Affiliations:** Department of Radiation Oncology, University of Texas Southwestern Medical Center, Dallas, TX 75390, United States of America

**Keywords:** diffusion-weighted imaging, distortion correction, multimodal registration, implicit neural representation, landmark matching

## Abstract

*Objective.* Geometric distortions in diffusion-weighted imaging (DWI) compromise accurate tumor delineation and spatial localization, limiting its utility in radiation therapy planning and response monitoring. These distortions can be corrected through multimodal registration between distorted DWI and undistorted anatomical images, while conventional mutual information-based optimization often fails due to local minima and produces non-smooth, physically implausible deformations. *Approach.* This study proposes a landmark matching B-spline implicit neural representation framework for DWI distortion correction. The method integrates anatomical correspondences from a foundation landmark matching model with B-spline parameterized deformation fields to overcome local minima inherent in mutual information optimization. The framework employs Fourier-encoded multi-layer perceptrons to model B-spline deformation fields while ensuring physically plausible transformations, enabling robust multimodal registration between distorted DWI and anatomical references. *Main results.* Evaluation on brain and abdominal datasets demonstrated superior performance compared to established methods. The proposed approach achieved average Dice coefficients of 0.919 ± 0.038 (brain) and 0.926 ± 0.032 (abdomen), significantly outperforming all baseline methods. On simulated data, our method achieved an average PSNR of 25.912 ± 3.148 dB, NCC of 0.911 ± 0.137, and SSIM of 0.888 ± 0.107, the best among all methods. *Significance.* By combining the regularization properties of B-spline parameterization with the cross-modal matching capabilities of foundation models, our method achieves more accurate correction of geometric distortions in DWI, with the potential to enhance the precision of intra/post-radiotherapy assessment.

## 1. Introduction

Diffusion-weighted imaging (DWI) has become an essential tool in clinical practice, providing unique tissue contrast based on the restricted diffusion of water molecules. Applications range from early stroke detection (Schaefer *et al* 2000) to tumor characterization (Padhani 2009) and treatment response assessment (Koh and Collins 2007). Single-shot Echo-planar imaging (SS-EPI), the standard acquisition technique for DWI, enables rapid data collection but introduces geometric distortions due to magnetic field inhomogeneities (Jezzard and Balaban 1995) and eddy currents (Reese *et al* 2003). These distortions manifest as pixel shifts along the phase-encoding direction, compromising anatomical accuracy and spatial localization for quantitative analysis (Bihan *et al* 2001). Importantly, these geometric distortions remain mostly consistent across all *b*-values within a single acquisition session. This consistency enables a practical correction strategy: estimate a deformation field for distortion correction from DWI (*b* = 0) and apply it to all higher *b*-value images (Hagmann *et al* 2006).

Several approaches exist for DWI distortion correction. Advanced acquisition techniques include multi-shot EPI (MS-EPI), which reduces distortions by segmenting k-space acquisition but suffers from motion sensitivity and prolonged scan time (Porter and Heidemann 2009, Tamada *et al* 2022). RESOLVE (readout-segmented EPI), a specific type of MS-EPI, improves geometric fidelity through segmented readout with navigator correction, though residual artifacts persist at air-tissue interfaces (Porter and Heidemann 2009, Xia *et al* 2016). Turbo spin-echo DWI (TSE-DWI) achieves minimal distortion using refocusing pulses but is limited by low SNR and long acquisition time (Yoshizako *et al* 2021). Simultaneous multi-slice (SMS) techniques accelerate acquisition through concurrent slice excitation, yet may introduce slice-leakage artifacts and g-factor noise penalties (Barth *et al* 2016, Lawrence *et al* 2022). TOPUP (Andersson *et al* 2003), widely used in FSL (Jenkinson *et al* 2012), requires additional reversed phase-encoding acquisitions to estimate susceptibility-induced distortions. Field-mapping techniques (Jezzard and Balaban 1995) directly measure inhomogeneities but similarly require extra acquisitions. Many such approaches require specially-designed sequences, which are not available on magnetic resonance imaging-guided linear accelerators (MR-LINACs) (Yuan *et al* 2022). When such specialized acquisitions are unavailable, image registration to undistorted anatomical references remains the most practical correction strategy (Lu *et al* 2011, Bhushan *et al* 2015, Schilling 2019).

The fundamental challenge lies in registering the distorted DWI image to an undistorted anatomical reference, typically a T1-weighted, T2-weighted, or FLAIR acquisition. This constitutes a multimodal registration problem, as DWI images and reference images exhibit different tissue contrasts. To circumvent the contrast variation challenges in deformable registration, Synb0-DisCo (Schilling 2019) synthesizes an undistorted b0 image from T1-weighted data using deep learning, effectively enabling single-modal registration-driven distortion correction. However, its training requires specially acquired readout-segmented EPI images without distortion as ‘ground truth’, which are rarely available in routine clinical protocols. For multimodal registration, previous registration approaches have relied heavily on mutual information (MI) as the similarity metric. Classical methods like symmetric normalization (SyN) (Avants *et al* 2008) employ diffeomorphic transformations with MI optimization, while recent learning-based approaches, including VoxelMorph (Balakrishnan *et al* 2019) and TransMorph (Chen *et al* 2022) , similarly adopt MI-based loss functions for training. Despite its widespread adoption, MI-based optimization presents well-documented limitations: multiple local minima, sensitivity to initialization, and tendency to produce non-smooth deformations when unconstrained (Pluim *et al* 2003). As a purely statistical metric, MI lacks higher-level semantic understanding of anatomical structures, often resulting in physically implausible solutions.

To address the limitations of MI-based optimization, recent advances have focused on developing models with enhanced cross-modal understanding. SynthMorph (Hoffmann *et al* 2021) pioneered contrast-invariant registration by training exclusively on synthetic data with arbitrary contrasts, forcing the network to learn features independent of specific imaging modalities. While this approach demonstrates significant improvements over traditional methods, it was trained solely on synthetically generated images with varying contrasts. Without using any real medical images for training, its clinical applicability and accuracy can be limited. The recently proposed MultiGradICON (Demir *et al* 2024) extends this paradigm through loss function randomization, explicitly training networks to handle arbitrary contrast combinations by randomly pairing input modalities with different loss functions during training. While this strategy proves particularly effective for multimodal scenarios, yielding better results in cross-contrast registration tasks, its training data did not include any DWI acquisitions and thus may not handle DWI-specific distortions well (Demir *et al* 2024). Besides, DINO-Reg (Song *et al* 2024) leverages self-supervised vision transformers to extract semantic features for registration, demonstrating that foundation models pre-trained on diverse visual data can capture anatomical correspondences beyond traditional intensity-based metrics. While DINO-Reg provides powerful semantic feature extraction, it was not specifically trained for multimodal medical image matching tasks. Therefore, employing a robust cross-modal feature matching model could effectively establish reliable anatomical correspondences to guide the registration process. MINIMA (Ren *et al* 2025), a recent development, can be particularly well-suited for this purpose, as it is designed specifically for cross-modal image matching and employs a data-driven approach that trains on diverse modality pairs from both natural and medical images. MINIMA’s multimodal specialization and proven performance across 19 cross-modal scenarios, including various medical imaging modalities, make it particularly suitable for establishing reliable correspondences in medical image registration.

However, while MINIMA provides robust cross-modal matching capabilities, directly estimating continuous, dense deformation fields from discrete correspondences remains challenging. Implicit neural representations (INRs) offer an elegant solution by parameterizing deformation fields as continuous functions through neural networks (Sitzmann *et al* 2020, Tancik *et al* 2020). When combined with B-spline basis functions (Rueckert *et al* 2002, Sideri-Lampretsa *et al* 2024), INRs can model physically plausible deformations that match the inherent characteristics of SS-EPI distortions. The combination of semantic guidance from multimodal matchers with the physically-motivated regularization of B-spline-parameterized INRs addresses both the correspondence and smoothness requirements for robust DWI distortion correction. Building upon these insights, we present a registration framework that synergistically combines multimodal landmark matching with B-spline parameterized INRs for DWI distortion correction. Our method leverages MINIMA to establish robust anatomical correspondences between DWI and reference anatomical images, providing reliable guidance in regions where MI alone struggles. These landmarks anchor the optimization of a continuous deformation field represented through an INR with Fourier positional encoding. Crucially, B-spline parameterization constrains the deformation to smooth, physically plausible transformations, preventing the overfitting common in unconstrained MI optimization.

Our contributions address the core challenges of DWI distortion correction: (1) integration of foundation model features with traditional similarity metrics, combining the precision of learned correspondences with the flexibility of intensity-based optimization; (2) B-spline parameterized INR that enforces physically motivated smoothness while preserving necessary local flexibility; (3) comprehensive validation on brain and abdominal datasets, demonstrating consistent improvements over state-of-the-art methods.

## 2. Methods

### 2.1. Problem formulation

Given a distorted diffusion-weighted image *m* at *b* = 0 (moving image) and an undistorted anatomical reference *f* (fixed image), we seek a deformation field $\phi: \mathbb{R}^3 \rightarrow \mathbb{R}^3$ that spatially aligns *m* with *f*. The warped image $m \circ \phi$ should exhibit maximal anatomical correspondence with *f* while maintaining smooth, physically plausible deformations. Once estimated from the DWI (*b* = 0), *φ* is applied to all *b*-values to correct geometric distortions across the entire DWI acquisition.

### 2.2. Overview

**Figure 1.**
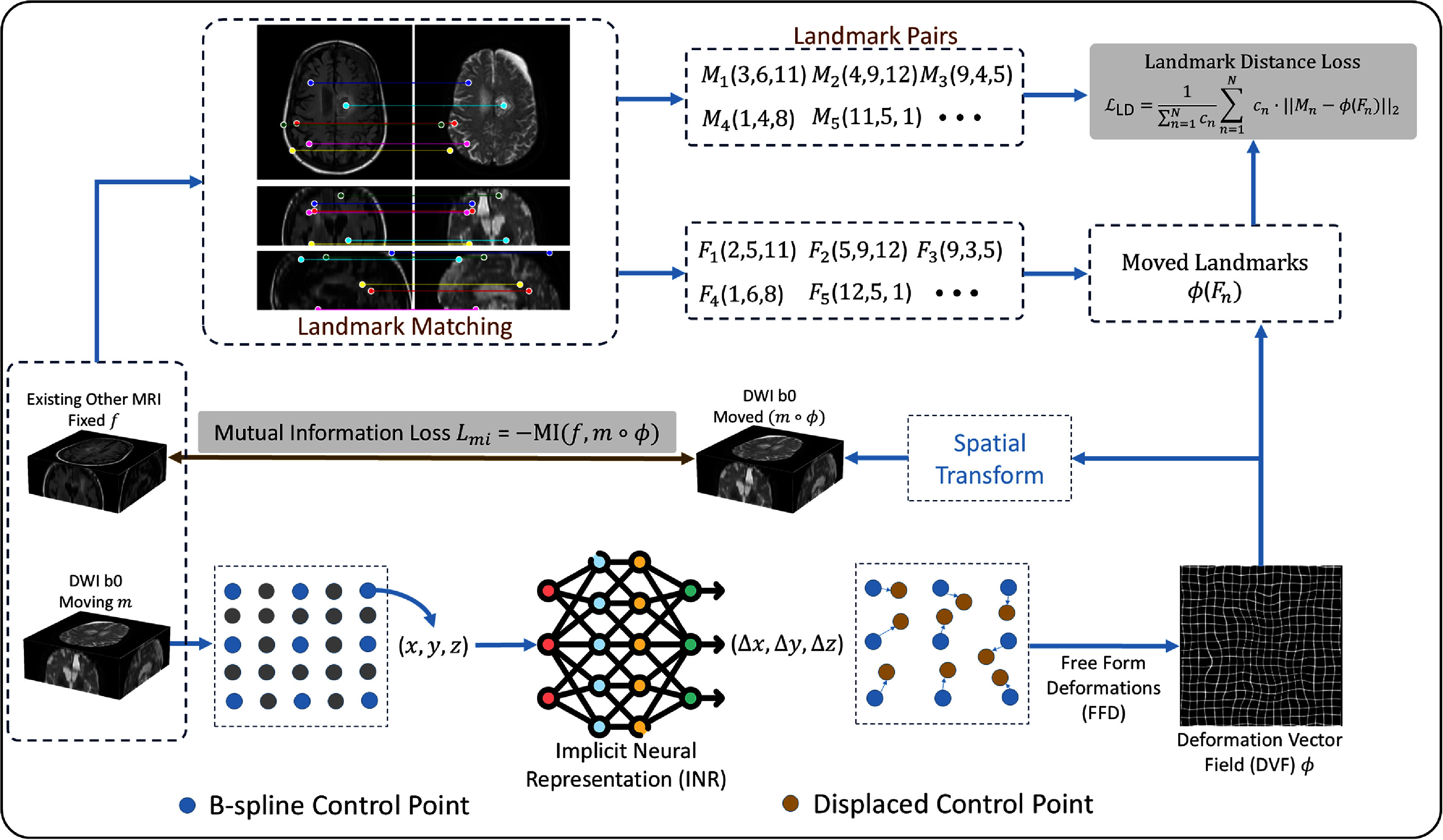
Overview of the landmark matching B-spline implicit neural representation (LMBS-INR) framework for DWI distortion correction. The method combines two complementary pathways: (top) a landmark matching stream that extracts anatomical correspondences between distorted DWI and undistorted reference images using pre-trained multimodal feature matchers across multiple orthogonal views; (bottom) a geometric deformation stream where B-spline control points are displaced through an INR with Fourier positional encoding. The deformation field *φ* is optimized using a composite loss comprising mutual information for intensity alignment and landmark distance for anatomical correspondence.

Figure 1 illustrates the landmark matching B-spline implicit neural representaion (LMBS-INR) framework, which integrates two complementary streams: (1) a geometric stream that predicts deformation fields through B-spline-parameterized INRs, and (2) a feature-matching stream that provides anatomical landmark supervision from pre-trained multimodal matchers. The synergy between learned correspondences and smooth deformation modeling addresses the fundamental limitations of conventional MI optimization.

### 2.3. Multi-view landmark extraction

We employ a multi-view strategy to extract comprehensive 3D anatomical correspondences. For each orthogonal view-axial, sagittal, and coronal-we extract 2D slices and apply feature matching using MINIMA (Ren *et al* 2025), a multimodal matcher built upon SuperPoint+LightGlue (Lindenberger *et al* 2023) and specifically trained for cross-modal image matching with demonstrated effectiveness in medical imaging applications. Unlike traditional single-modality feature matchers, MINIMA learns to extract imaging contrast-invariant semantic features by training on large-scale synthetic multimodal datasets encompassing diverse modality combinations, enabling it to establish reliable anatomical correspondences between modality pairs such as DWI and anatomical references even when they exhibit drastically different intensity patterns.

For axial slices at position *z*, the matcher identifies corresponding point pairs $\{(M_n^{(z)}, F_n^{(z)}, c_n^{(z)})\}_{n = 1}^{N_z}$ between the moving image (distorted DWI) and the fixed image (undistorted anatomical reference). Here, $M_n^{(z)}$ denotes the 2D coordinates of the *n*-th landmark on the moving image, $F_n^{(z)}$ denotes the 2D coordinates of the corresponding *n*-th landmark on the fixed image representing the same anatomical feature, $c_n^{(z)} \in [0, 1]$ represents the confidence score for this correspondence, and *n* indicates the matched point pairs. Each 2D correspondence is lifted to 3D by appending the slice coordinate *z*: $M_n = (M_{nx}, M_{ny}, z)$, $F_n = (F_{nx}, F_{ny}, z)$. This process repeats for sagittal (constant *x*) and coronal (constant *y*) views, yielding a comprehensive set of 3D landmarks $\{(M_i, F_i, c_i)\}_{i = 1}^{N}$ that capture anatomical correspondences across the entire volume with associated reliability measures, where *i* indicates the matched landmark pairs across all three views. The multi-view approach promotes more robust feature coverage, particularly in regions where single-view matching might fail due to partial volume effects, limited in-plane contrast, or large inter-slice motion.

### 2.4. B-spline parameterized INR

We parameterize the deformation field using a uniform cubic B-spline grid with control point spacing *d*. The B-spline representation provides two key advantages: (1) automatic regularization of deformation degrees of freedom through control point count, and (2) yielding a deformation field that is smooth. Rather than directly optimizing a dense displacement field, we predict control point displacements $\Delta p_i = (\Delta x_i, \Delta y_i, \Delta z_i)$ through an INR:

\begin{equation*} \Delta p_i = g_\theta\left(\gamma\left(p_i\right)\right),\end{equation*}

where *g*_*θ*_ is a multi-layer perceptron with parameters *θ*, and $\gamma(\cdot)$ represents Fourier feature encoding. The network architecture employs a five-layer fully connected structure with 512 hidden units per layer using ReLU activation functions. The Fourier feature encoding is defined as:

\begin{equation*} \gamma\left(p\right) = \left[\sin\left(2\pi \mathbf{B}p\right), \cos\left(2\pi \mathbf{B}p\right)\right]^T, \quad \mathbf{B} \sim \mathcal{N}\left(0, \sigma^2\right),\end{equation*}

where matrix **B** is a randomly initialized frequency matrix, typically sampled from a Gaussian distribution $\mathcal{N}(0, \sigma^2)$. The Fourier encoding enables the network to learn high-frequency spatial variations more effectively by lifting the input coordinates to a higher-dimensional feature space, crucial for capturing local details in SS-EPI distortions. Displaced control points $\tilde{p}_i = p_i + \Delta p_i$ induce a continuous deformation field through cubic B-spline interpolation:

\begin{equation*} \phi\left(x\right) = x + \sum_{i} \Delta p_i \cdot \beta^3\left(||x - p_i||/d\right),\end{equation*}

where *β*^3^ denotes the cubic B-spline basis function. The inherent properties of B-splines ensure smoothness and continuity of the deformation field, with each control point only affecting its local neighborhood. In our implementation, we adopt a convolution-based free-form deformation (FFD) framework for efficient B-spline interpolation computation (Rueckert *et al* 2002, Sideri-Lampretsa *et al* 2024). Control point displacements are upsampled to dense deformation fields through separable convolution operations, utilizing precomputed cubic B-spline kernels with multi-channel convolution followed by output reshuffling to obtain the final deformation field. This convolution-based approach is more efficient than point-wise evaluation of B-spline basis functions, particularly for large-scale 3D medical images. The control point spacing *d* is a critical parameter balancing deformation flexibility and regularization: smaller *d* values allow more localized deformations but may lead to overfitting, while larger *d* values impose stronger smoothness constraints. Based on parameter sensitivity analysis (section 3.6), we set *d* to 30 voxels.

### 2.5. Optimization objective

The network parameters *θ* are optimized through a composite loss function that balances intensity similarity and landmark correspondence. This multi-objective optimization strategy combines intensity-based global alignment with feature-based local accuracy.

**MI Loss:** To handle the multimodal nature of DWI-to-reference registration, we maximize the MI between fixed and warped images:

\begin{equation*} \mathcal{L}_{mi} = -MI\left(f, m \circ \phi\right) = -\sum_{i,j} p\left(i,j\right) \log \frac{p\left(i,j\right)}{p_f\left(i\right)p_m\left(j\right)},\end{equation*}

where $p(i,j)$ is the joint probability distribution, and $p_f(i)$, $p_m(j)$ are the marginal distributions of fixed and moving images respectively. Following previous work (Thévenaz and Unser 2000, Sideri-Lampretsa *et al* 2024), we compute these distributions using Parzen window density estimation. This non-parametric approach avoids assumptions about intensity distributions, making it robust to different imaging protocols.

**Landmark loss:** The landmark correspondences provide explicit anatomical guidance, particularly valuable in regions of low contrast or ambiguous intensity relationships. Each correspondence is weighted by its confidence score to account for matching reliability:

\begin{equation*} \mathcal{L}_\mathrm {LD} = \frac{1}{\sum_{n = 1}^{N} c_n} \sum_{n = 1}^{N} c_n \cdot ||M_n - \phi(F_n)||_2,\end{equation*}

where $c_n \in [0,1]$ is the confidence score from the MINIMA matcher, $\phi(F_n)$ denotes the landmarks from the fixed image transformed by the deformation field *φ* (as the deformation field is defined on the fixed image grid and points to the moving image), and *M_n_* denotes the corresponding landmarks on the moving image. The loss function measures the spatial distance between the transformed landmarks from the fixed image and the landmarks from the moving image via the L2 norm, encouraging the deformation field to accurately align corresponding anatomical locations. The confidence weighting mechanism provides an adaptive approach to handling matching uncertainty: the confidence score *c_n_* is automatically computed by MINIMA based on feature descriptor similarity and geometric consistency, with high-confidence matches (typically corresponding to regions with distinctive anatomical features, such as ventricular corners or organ boundaries) having greater influence on optimization, while low-confidence matches are automatically down-weighted to prevent potential incorrect matches from misleading the registration process. The normalization factor $1/\sum_{n = 1}^{N} c_n$ ensures that the magnitude of the landmark loss is independent of the total confidence sum, maintaining a balanced contribution along with the MI loss. The total loss combines these terms with an empirically determined weight:

\begin{equation*} \mathcal{L}_\mathrm {total} = \mathcal{L}_\mathrm {mi} + \lambda_\mathrm {LD} \mathcal{L}_\mathrm {LD},\end{equation*}

where $\lambda_\mathrm {LD} = 2.0$ (section 3.6) balances the contribution of landmark guidance relative to MI. This weight was determined through a grid search to achieve optimal local anatomical accuracy while maintaining global intensity alignment.

### 2.6. Data preprocessing

Before applying the proposed registration framework, input images undergo standardized preprocessing to ensure optimization stability and consistency across different scanning protocols. The preprocessing pipeline consists of the following steps:

**Intensity normalization:** *Z*-score normalization is applied independently to the fixed image (anatomical reference) and the moving image (distorted DWI) for intensity standardization. For each image, the mean *µ* and standard deviation *σ* of intensity values are computed, and each voxel intensity *I* is transformed to $(I-\mu)/\sigma$. This normalization approach standardizes intensity range variations across different acquisition protocols and scanners, making MI computation more stable. It is important to emphasize that this normalization is applied solely during the registration optimization phase to estimate the deformation field. Once the deformation field *φ* is obtained, the field is applied directly to the original, unnormalized DWI images (across all *b*-values), thereby preserving the original intensity values of the images.

**Spatial resampling:** The moving image (distorted DWI) is resampled to the coordinate system of the fixed image (anatomical reference). Resampling uses trilinear interpolation to ensure that both images have identical voxel spacing, image dimensions, and spatial coordinate systems. This step is crucial for multi-view landmark extraction, as the MINIMA matcher requires extracting corresponding 2D slices from image pairs with consistent spatial resolution. It is worth noting that in our datasets, the DWI images and anatomical reference images are acquired within the same scanning session with identical patient setup and scanner geometry. Therefore, the two images already have good initial spatial alignment, eliminating the need for additional rigid pre-alignment steps. This single-session acquisition strategy is common in clinical practice and simplifies the preprocessing workflow.

## 3. Results

### 3.1. Experimental setup

#### 3.1.1. Datasets

Four distinct datasets were employed to comprehensively evaluate the proposed LMBS-INR framework. The first dataset (simulated distortion dataset) comprised 75 multimodal brain MRI cases, where FLAIR images underwent synthetic deformation with a maximum displacement of 20 voxels along the phase encoding direction to establish ‘ground truth’ for quantitative validation. For each case, the T1 or T2 images were used as the anatomical references to correct the distorted FLAIR images. The second dataset is derived from the publicly available ON-Harmony (Oxford–Nottingham Harmonization) resource (Warrington 2025), a multi-center, multi-modal dataset for MRI harmonization research. This dataset includes 20 volunteers with both DWI and T2-weighted structural imaging. Critically, the DWI acquisitions in this dataset include *b* = 0 images in both phase-encoding directions (PA and AP), enabling distortion correction using FSL’s TOPUP method as a reference. While TOPUP requires additional acquisition of reverse phase-encoded images, increasing scan time, our proposed method uses only routinely acquired structural MR images as anatomical references, without requiring additional specialized reverse phase-encoding scans. We use the TOPUP-processed results as a near-distortion-free reference standard to evaluate the consistency of our method with the TOPUP method. For clinical validation, we collected DWI images from 10 brain cases, which were paired with FLAIR references (dataset 3). In addition, we collected DWI images from 9 abdominal cases, which were paired with T2-SPIR references (dataset 4). For datasets 3 and 4, all DWI acquisitions employed parallel imaging with an acceleration factor of 2.5. The DWI sequences included multiple *b*-values acquired using standard SS-EPI protocols. Since geometric distortions remain nearly identical across all *b*-values within a single acquisition, we estimate the deformation field solely from the DWI (*b* = 0) and apply it to all *b*-value images for distortion correction.

#### 3.1.2. Implementation details

The LMBS-INR architecture utilized a five-layer MLP with 512 hidden units per layer and ReLU activations for the first four layers. B-spline control point spacing was set to 30 voxels based on parameter optimization (section 3.6). Landmark matching employed MINIMA for its superior cross-modal performance. The landmark loss weight $\lambda_\mathrm {LD}$ was fixed at 2.0 following sensitivity analysis. Optimization proceeds using the Adam optimizer (Kingma and Ba 2015) with learning rate 10^−4^ and momentum parameters $\beta_1 = 0.9$, and $\beta_2 = 0.999$. Training runs for 150 epochs. All experiments were conducted on NVIDIA RTX 4090 GPUs with 24GB memory. The code is available on the GitHub repository: https://github.com/Kent0n-Li/LMBS-INR.

#### 3.1.3. Evaluation metrics and statistical analysis

Registration accuracy was quantified using multiple complementary metrics: peak signal-to-noise ratio (PSNR) for intensity fidelity, normalized cross-correlation (NCC) for structural alignment, and structural similarity index (SSIM) for perceptual quality. For anatomical accuracy assessment, we computed Dice coefficients on manually delineated regions of interest (ROIs) selected based on boundary visibility in the reference images. Statistical significance was assessed using Wilcoxon signed-rank tests. All reported p-values represent comparisons against our proposed method, with significance threshold set at *p* < 0.05.

#### 3.1.4. Baseline methods

Seven state-of-the-art registration methods served as baselines: SyN (Avants *et al* 2008) representing classical diffeomorphic registration, VoxelMorph (Balakrishnan *et al* 2019) for unsupervised deep registration, TransMorph (Chen *et al* 2022) as a Transformer-based approach, SynthMorph (Hoffmann *et al* 2021) for contrast-invariant registration, MultiGradICON (Demir *et al* 2024) for multimodal registration, DINO-Reg (Song *et al* 2024) leveraging self-supervised vision transformers for semantic registration, and CorrMLP (Meng *et al* 2024) as a correlation-aware multi-layer perceptron approach for deformable registration. All baselines were implemented using the official code with recommended hyperparameters.

### 3.2. Performance on the simulated distortion dataset

**Figure 2.**
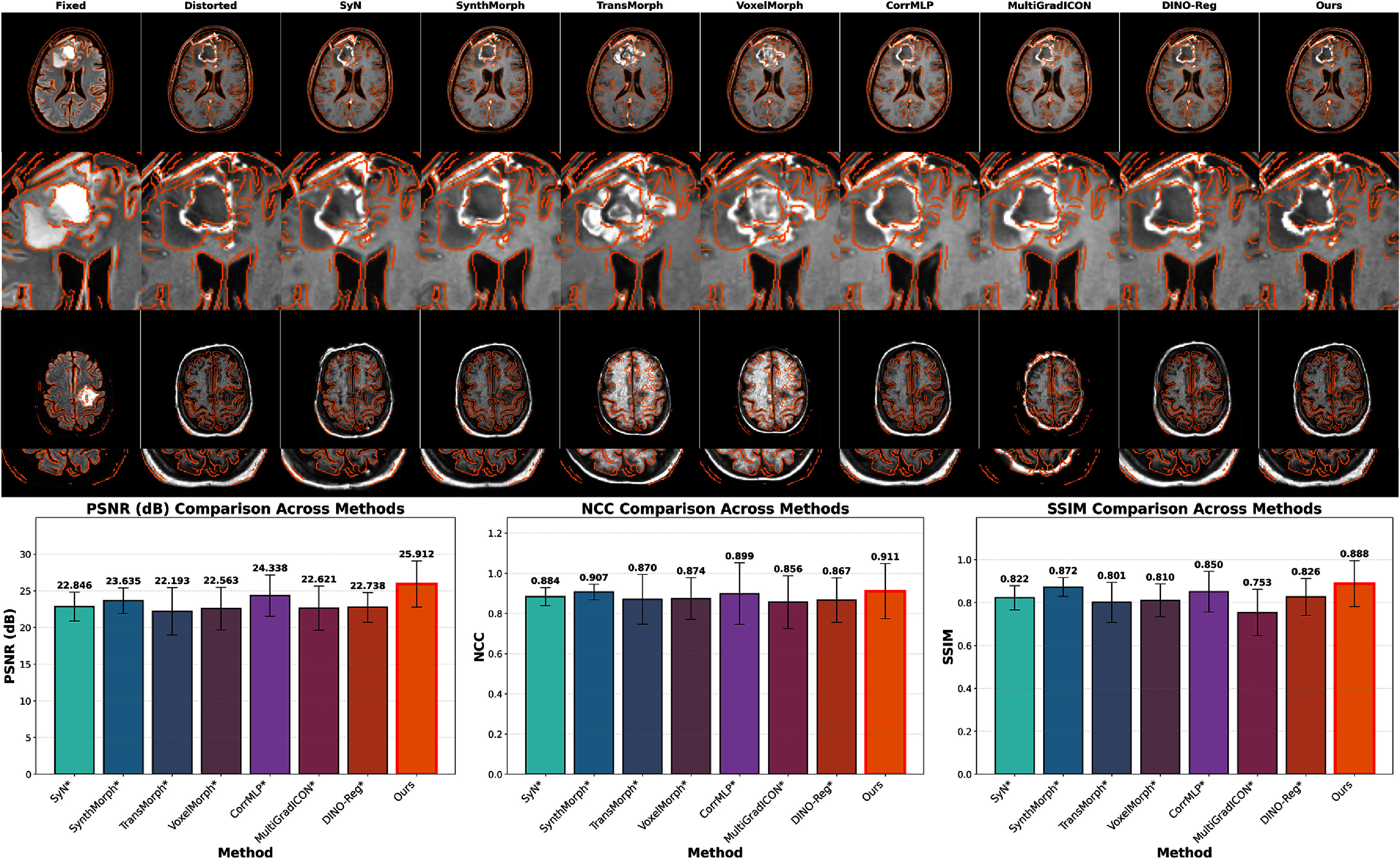
Evaluation on the simulated distortion dataset. Top rows display representative registration results across methods (rows 1 and 3 show two different cases, rows 2 and 4 show corresponding zoomed-in regions), with orange contours extracted using Canny edge detection (Canny 1986) from the fixed image. Bottom row presents quantitative metrics (PSNR, NCC, and SSIM) across all 75 test cases. The proposed LMBS-INR method (rightmost) consistently achieves superior performance with statistically significant improvements (all p-values $ < $ 0.05, Wilcoxon signed-rank test). Error bars represent standard deviations.

Figure 2 presents evaluation results on the simulated distortion dataset with known ‘ground-truth’ deformations. The proposed LMBS-INR method achieved an average PSNR of 25.912 dB, demonstrating significant improvement over the second-best method (SynthMorph at 23.635 dB) by 2.277 dB (*p* < 0.05). Similarly, NCC values reached 0.911 for our approach, while SynthMorph, designed for cross-contrast registration, achieved 0.907. SSIM metrics followed a consistent pattern, with LMBS-INR achieving 0.888 versus 0.872 for SynthMorph. DINO-Reg, despite leveraging self-supervised vision transformers, achieved an average PSNR of 22.738 dB, NCC of 0.867, and SSIM of 0.826, underperforming SynthMorph. MultiGradICON showed notably poor performance with a 0.753 SSIM, likely due to the significant domain gap between its pre-training data and our MR data.

These quantitative improvements are clearly reflected in the visual results, as demonstrated in the upper panels of figure 2. While baseline methods exhibit residual misalignments particularly in periventricular regions and cortical boundaries, LMBS-INR achieves accurate anatomical correspondence throughout the brain volume. The orange contour overlays clearly illustrate superior boundary alignment, with our method closely matching the ‘ground-truth’ deformation. Detailed error analysis revealed that registration failures in baseline methods primarily concentrated in two critical regions: (1) skull regions where the intensity relationship between fixed and distorted images is reversed, creating challenges for MI-based methods; (2) tumor regions where VoxelMorph and TransMorph produced inappropriate internal structural deformations due to overfitting, which is unacceptable for medical image analysis as it corrupts critical pathological information for diagnosis and treatment planning. In contrast, our landmark-guided approach effectively avoids these failure modes by providing reliable anatomical anchors, preserving structural integrity by correcting geometric distortions.

**Figure 3.**
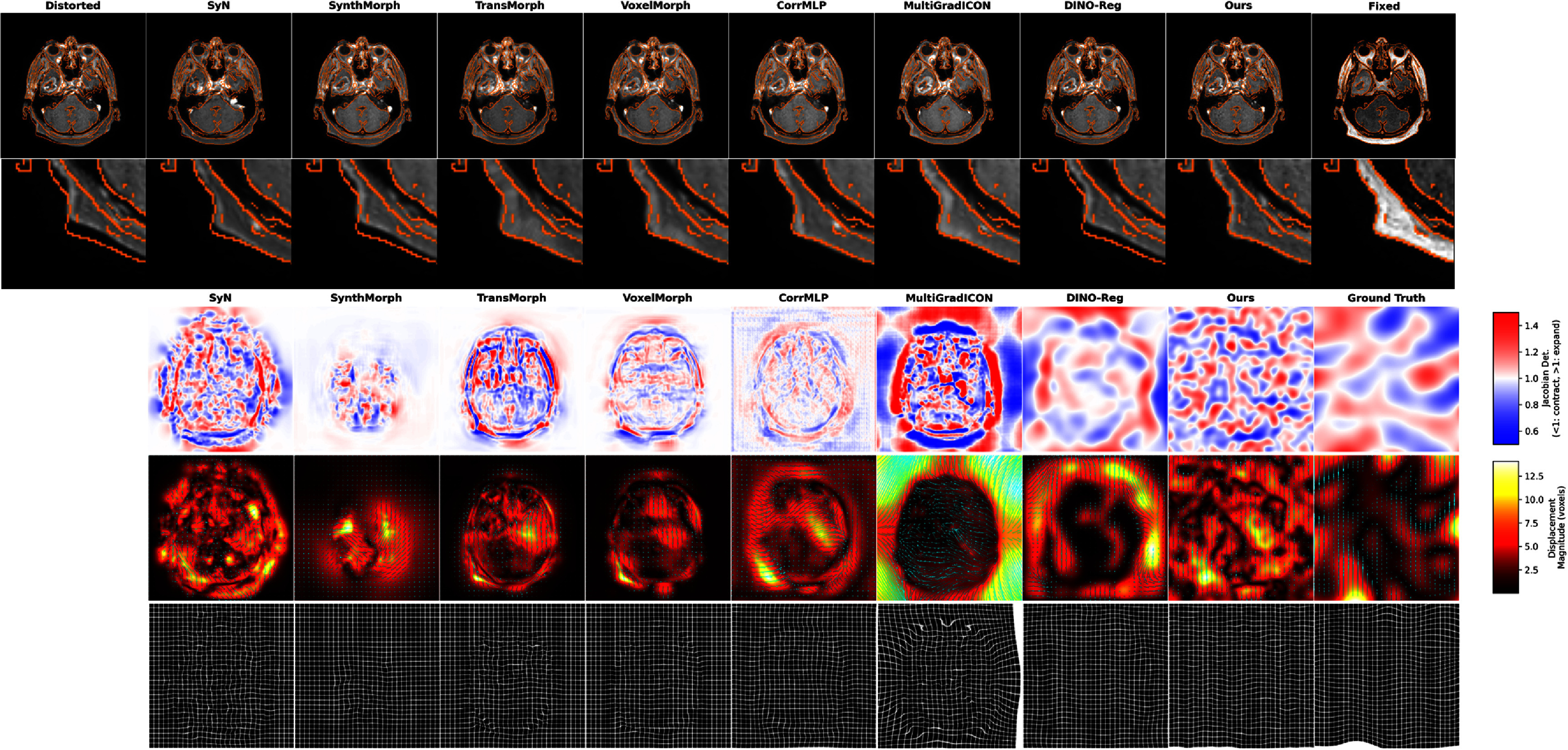
Visualization of deformation fields produced by different methods on the simulated distortion dataset. The first row displays the fixed image, distorted image, and registered images using different methods, with orange contours of the fixed image extracted by Canny edge detection and overlaid on different images. The second row shows zoomed-in regions of the first row. The third row shows Jacobian determinant maps with color coding indicating local volume changes of the deformation: red indicates expansion, blue indicates contraction. The fourth row displays the deformation field’s direction vectors and displacement magnitude, and the fifth row shows grid deformation visualization revealing local deformation patterns.

To further analyze the characteristics of deformation fields produced by different methods, figure 3 provides a detailed visualization of the deformation fields. The first two rows show visual comparison results with zoomed-in regions, where it can be seen that our method’s edges align better with the Canny-extracted fixed image edges than other methods. The third row’s Jacobian determinant color-coded maps reveal the local volume changes of the deformation: our LMBS-INR method shows smooth and physiologically plausible expansion-contraction patterns, with Jacobian values closer to the ‘ground truth’. In contrast, methods such as TransMorph and MultiGradICON exhibit extreme Jacobian values (deep red or blue regions) in certain areas, indicating unreasonable local compression or expansion. The fourth row displays the deformation field’s direction vectors and displacement magnitude, clearly showing the local displacement patterns estimated by each method, with our method’s displacement magnitude closest to the ‘ground truth’. The fifth row’s grid deformation visualization further confirms that grid deformations produced by our LMBS-INR method are smooth and regular, while other methods show varying degrees of grid distortion and folding, with larger deviations from the ‘ground truth’. It is worth emphasizing that all evaluated registration methods (including our LMBS-INR) are fully unconstrained 3D registration algorithms that allow free deformation in all spatial directions, not just the phase encoding direction. Therefore, from the visualization results in the fourth and fifth rows, we can see that the registration results of our LMBS-INR are more consistent with physical plausibility.

### 3.3. Comparison with TOPUP method

**Figure 4.**
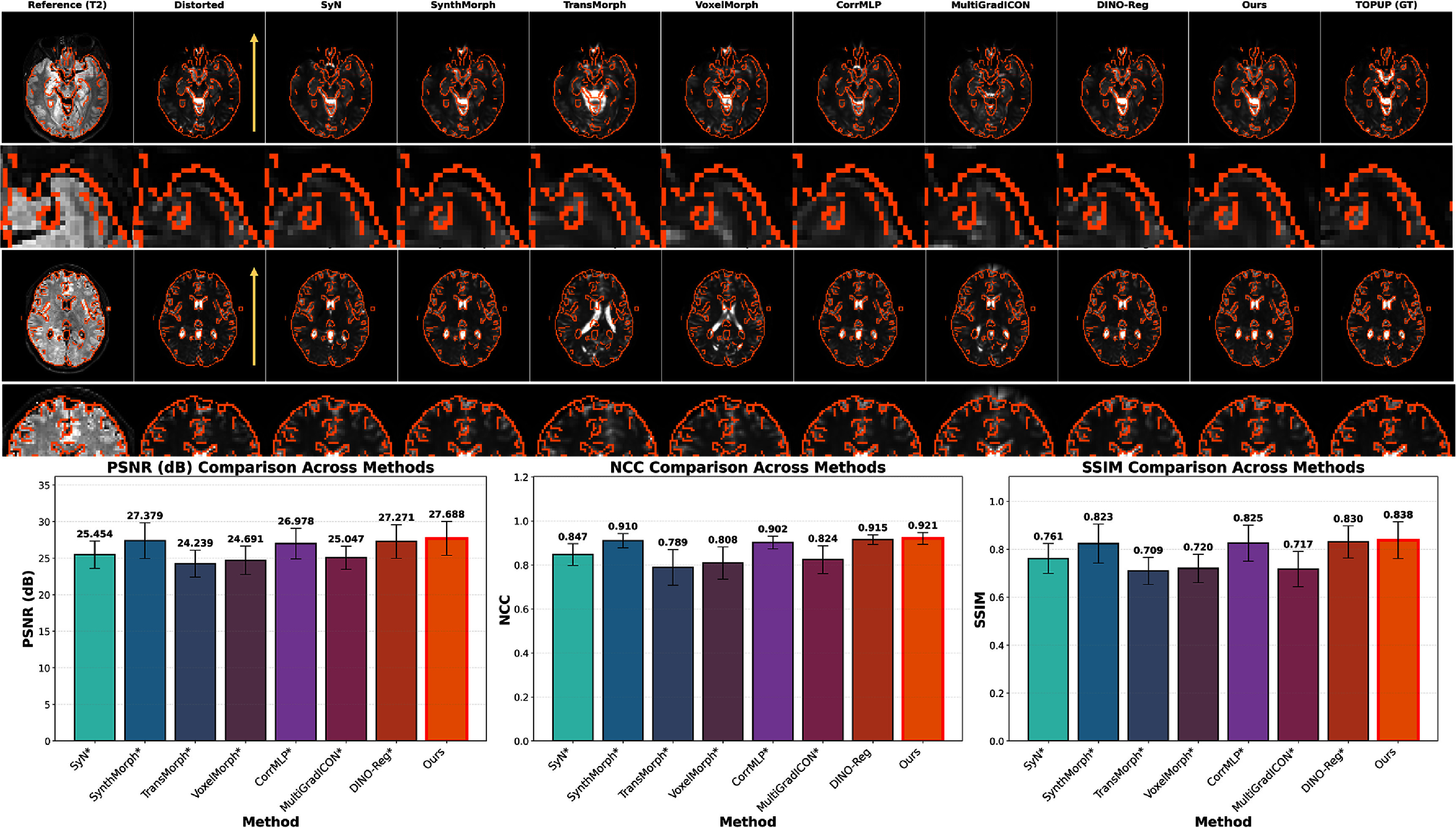
Comparison results with the TOPUP method. Upper panels show correction results for two representative cases (rows 1 and 3), with rows 2 and 4 displaying corresponding zoomed-in views of critical anatomical regions, showcasing alignment details of ventricular boundaries and cortical structures. Yellow arrows indicate the phase encoding direction. Bottom panel presents quantitative evaluation metrics (PSNR, NCC, and SSIM) across all 20 subjects from the ON-Harmony dataset, using TOPUP correction results as reference. Error bars represent standard deviations. Asterisks (*) indicate statistically significant differences compared to the proposed method (*p* < 0.05, Wilcoxon signed-rank test).

TOPUP is a widely used distortion correction method that requires additional acquisition of reverse phase-encoded images, utilizing bi-directional images (AP/PA, for instance) to estimate the distortion field. To further validate the effectiveness of our method, we compared it with the TOPUP method on the ON-Harmony dataset, using TOPUP’s distortion correction results as ‘ground truth’. Our method and other baseline methods use routinely acquired T2-weighted structural images as anatomical references to directly correct DWI distortions (phase-encoded along the AP direction). Figure 4 presents visual and quantitative comparison results on representative cases. In the visual assessment (upper panels of figure 4), the correction results produced by our method are very close to the TOPUP reference, demonstrating excellent alignment in critical anatomical regions such as ventricular boundaries and cortical structures. Although the zoomed-in regions in rows 2 and 4 of figure 4 show that our method’s boundaries have approximately one-voxel differences in some areas, the overall alignment is very good, while other baseline methods (particularly CorrMLP and MultiGradICON) exhibit substantially larger deviations. Quantitative evaluation (bottom panel of figure 4) further confirms these observations. Using TOPUP-corrected images as reference, our LMBS-INR method achieved the highest average PSNR of 27.688, NCC of 0.921, and SSIM of 0.838 among all evaluated methods. In contrast, DINO-Reg, as the second-best method, achieved an average PSNR of 27.271, NCC of 0.915, and SSIM of 0.830. These results demonstrate that our proposed registration-based method can achieve distortion correction performance close to the TOPUP method without requiring additional specialized reverse phase-encoding scans.

### 3.4. Clinical DWI correction results

**Figure 5.**
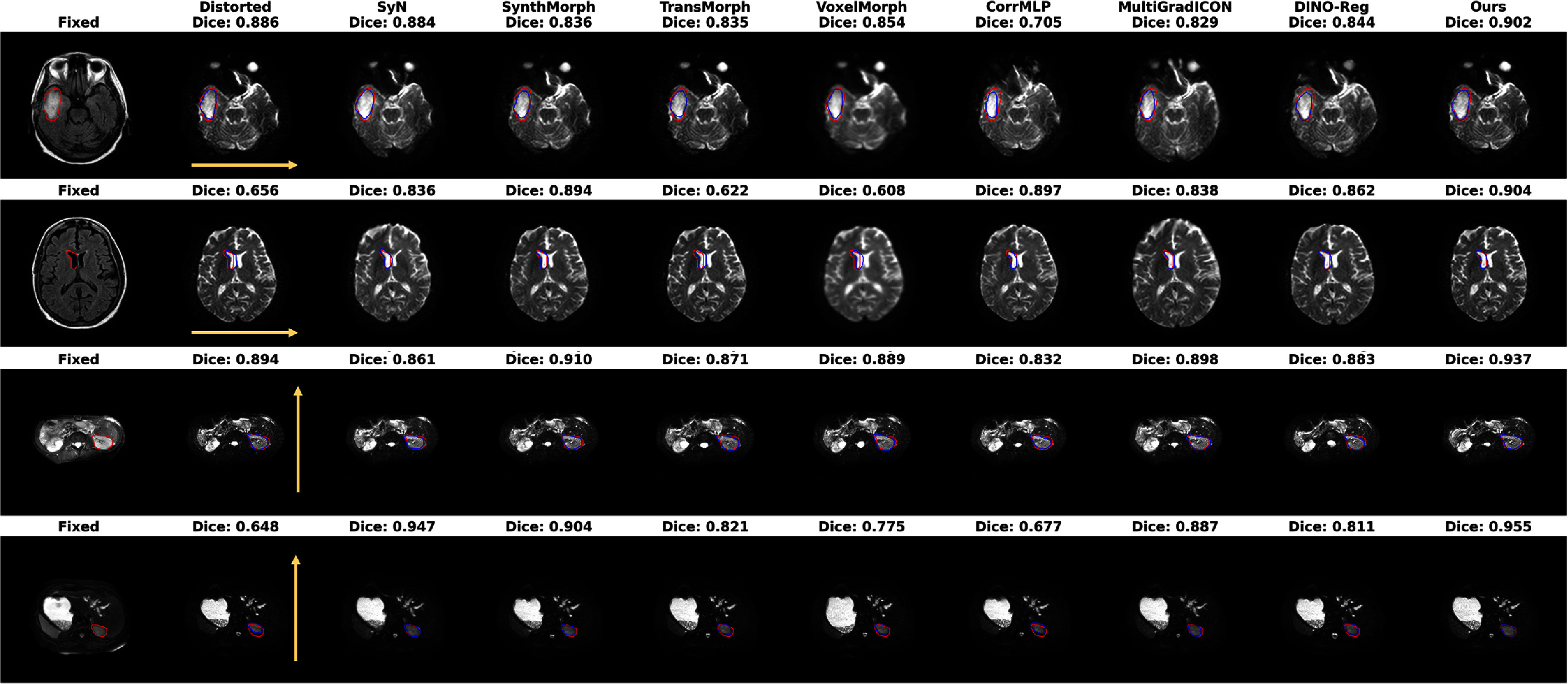
ROI-based evaluation on the clinical DWI data. Upper two rows show brain cases and lower two rows show abdominal cases. Red contours represent ROIs delineated on the fixed anatomical references (FLAIR for brain, and T2-SPIR for abdomen), while blue contours show corresponding ROIs on the registered DWI images from different methods. Yellow arrows indicate the phase encoding direction. Dice coefficients quantify the overlap between red and blue ROIs, with higher values indicating better geometric distortion correction. The proposed method achieves superior anatomical alignment across all evaluated structures.

While simulated distortion data provides controlled evaluation conditions, real clinical DWI data may exhibit more diverse characteristics. We therefore evaluated our method on clinical DWI datasets to validate its applicability. Since clinical DWI lacks ‘ground-truth’ deformation fields, we employed ROI-based Dice coefficient evaluation for quantitative assessment. ROIs were carefully delineated on anatomical reference images (FLAIR for brain, and T2-SPIR for abdomen), selecting regions with clear boundaries to minimize annotation uncertainty. The Dice coefficient between corresponding ROIs on the reference and registered DWI images quantifies registration accuracy, with higher values indicating better geometric distortion correction. Figure 5 presents ROI-based evaluation on representative clinical DWI cases. In brain cases (upper two rows), our method achieved Dice coefficients of 0.902 and 0.904 for the ROI-delineated regions including tumor areas and lateral ventricles, demonstrating the highest scores among all evaluated methods. The second brain case with a lateral ventricle ROI particularly highlights the performance differences: while the distorted baseline shows severe compression (Dice: 0.656), our method achieves nearly perfect alignment (Dice: 0.904), substantially outperforming SynthMorph (0.894) and other approaches. For the abdominal cases (lower two rows), our method achieves the highest Dice scores of 0.937 and 0.955, with the best overlap between red and blue contours indicating accurate correction of critical tissue distortions.

**Figure 6.**
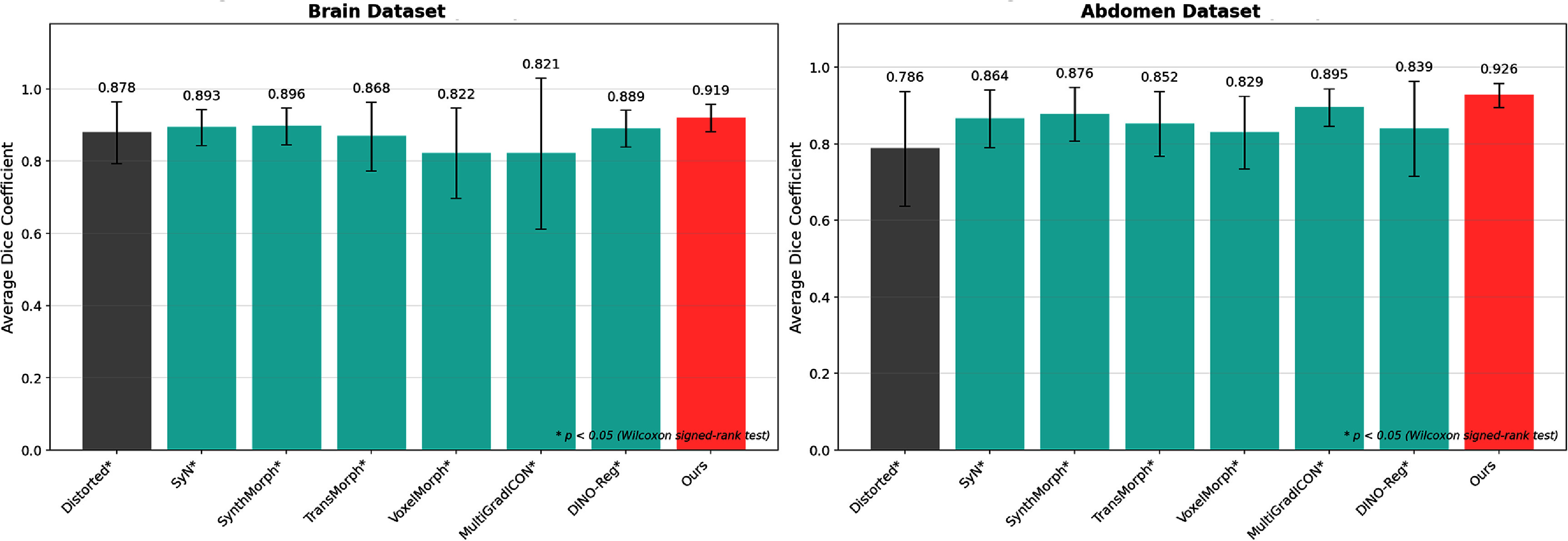
Average Dice coefficients across the brain (10 cases) and abdominal (9 cases) datasets. Asterisks (*) indicate statistically significant differences compared to the proposed method (*p* < 0.05, Wilcoxon signed-rank test). The red bars highlight our LMBS-INR approach achieving 0.919 (brain) and 0.926 (abdomen) on the average Dice, significantly outperforming all baseline methods.

Figure 6 presents the average Dice coefficients across all clinical cases, where the proposed method achieved 0.919 for brain and 0.926 for abdominal datasets, representing statistically significant improvements (*p* < 0.05, Wilcoxon signed-rank test) over all baseline methods. The distorted baseline images showed poor anatomical correspondence with average Dice scores of only 0.878 (brain) and 0.786 (abdomen), highlighting the severity of SS-EPI-induced geometric distortions. SyN, a diffeomorphic registration algorithm based on variational principles proposed in the ANTs toolkit (Avants 2009), has demonstrated excellent accuracy and topological preservation in numerous brain imaging comparison tasks. This explains SyN’s competitive performance, achieving average Dice of 0.893 in the brain dataset, ranking third after our method and SynthMorph. However, SyN’s advantage diminishes in abdominal imaging (Dice: 0.864), where the larger deformations may exceed its optimization capabilities. DINO-Reg, despite leveraging self-supervised vision transformers, achieved only 0.889 (brain) and 0.839 (abdomen), demonstrating that general-purpose features alone are insufficient for addressing DWI-specific distortions.

**Figure 7.**
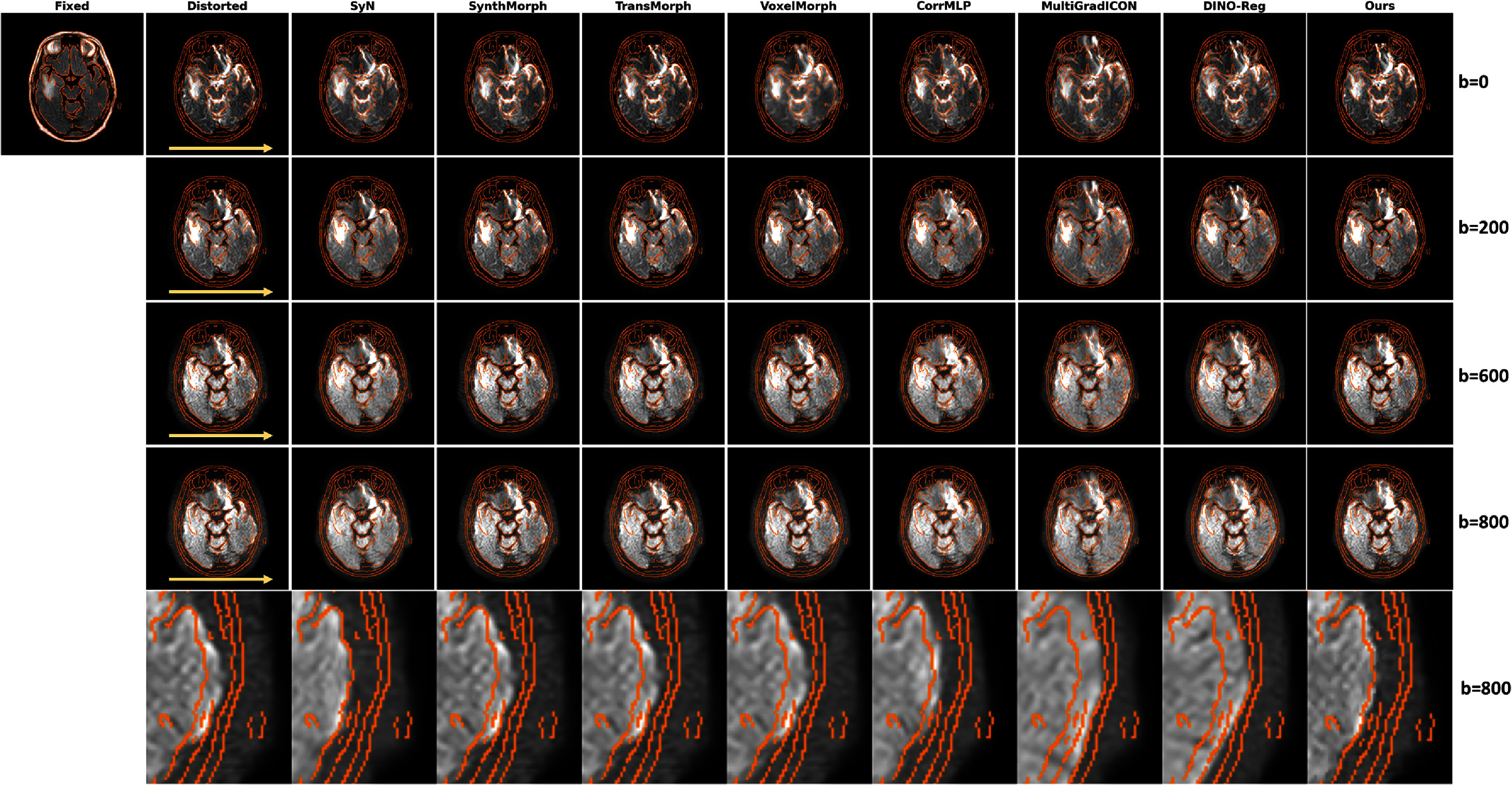
Multi-*b*-value registration results on brain DWI. Rows represent different *b*-values (*b* = 0, *b* = 200, *b* = 600, and *b* = 800) with deformation fields estimated from *b* = 0 images and applied across all diffusion weightings. Orange contours extracted using Canny edge detection on the FLAIR reference highlight anatomical boundaries. Yellow arrows indicate the phase encoding direction.

**Figure 8.**
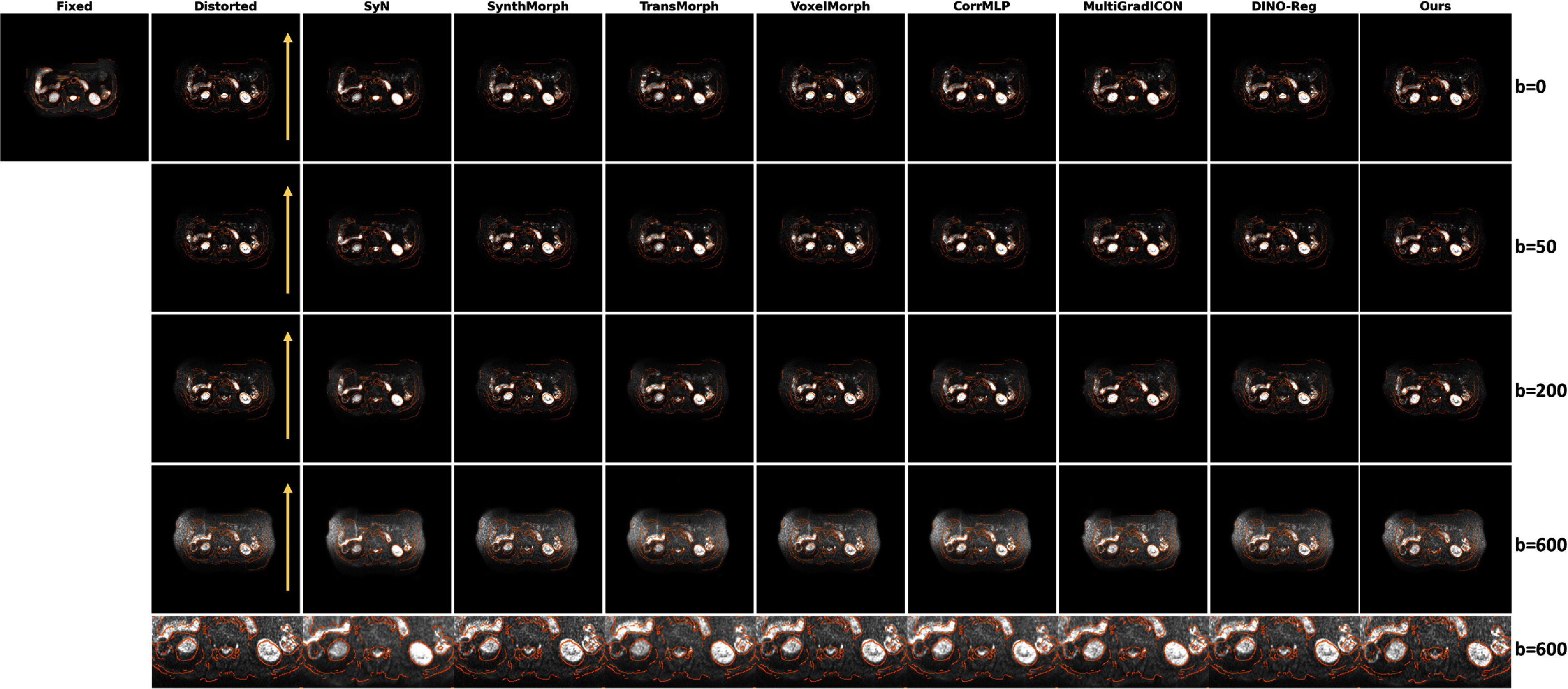
Abdominal DWI registration across multiple *b*-values. Rows represent different *b*-values (*b* = 0, *b* = 50, *b* = 200, and *b* = 600) with deformation fields estimated from *b* = 0 images and applied across the diffusion spectrum. Orange contours extracted using Canny edge detection on the T2-SPIR reference demonstrate anatomical alignment quality. Yellow arrows indicate the phase encoding direction.

The multi-*b*-value evaluation validated our approach of estimating deformation fields from *b* = 0 images and applying them across all diffusion weightings. Figure 7 demonstrates the brain DWI registration results across multiple *b*-values (*b* = 0, *b* = 200, *b* = 600, and *b* = 800). The orange contours, extracted via Canny edge detection (Canny 1986) from the FLAIR reference, provide clear visualization of anatomical boundaries. The proposed method maintains excellent alignment across all *b*-values, with the ventricle boundaries and cortical structures well matching the reference contours. In contrast, baseline methods show varying degrees of misalignment: SyN exhibits slight deviations in the occipital lobe region, while other baseline models (SynthMorph, TransMorph, VoxelMorph, MultiGradICON, and DINO-Reg) all demonstrate poor registration performance in the lateral brain regions as shown in the magnified view (bottom row). Our method achieves superior gray-white matter boundary preservation throughout. Figure 8 presents corresponding results for abdominal DWI across *b*-values (*b* = 0, *b* = 50, *b* = 200, and *b* = 600). The challenges in abdominal imaging are immediately apparent: larger organ deformations. Despite this complexity, LMBS-INR achieves remarkable alignment with the T2-SPIR reference (orange contours). The liver boundaries, kidney outlines, and spleen margins are accurately restored across all *b*-values. Other baseline models including SynthMorph, VoxelMorph, and DINO-Reg all exhibit clear misalignment.

### 3.5. Ablation study

**Figure 9.**
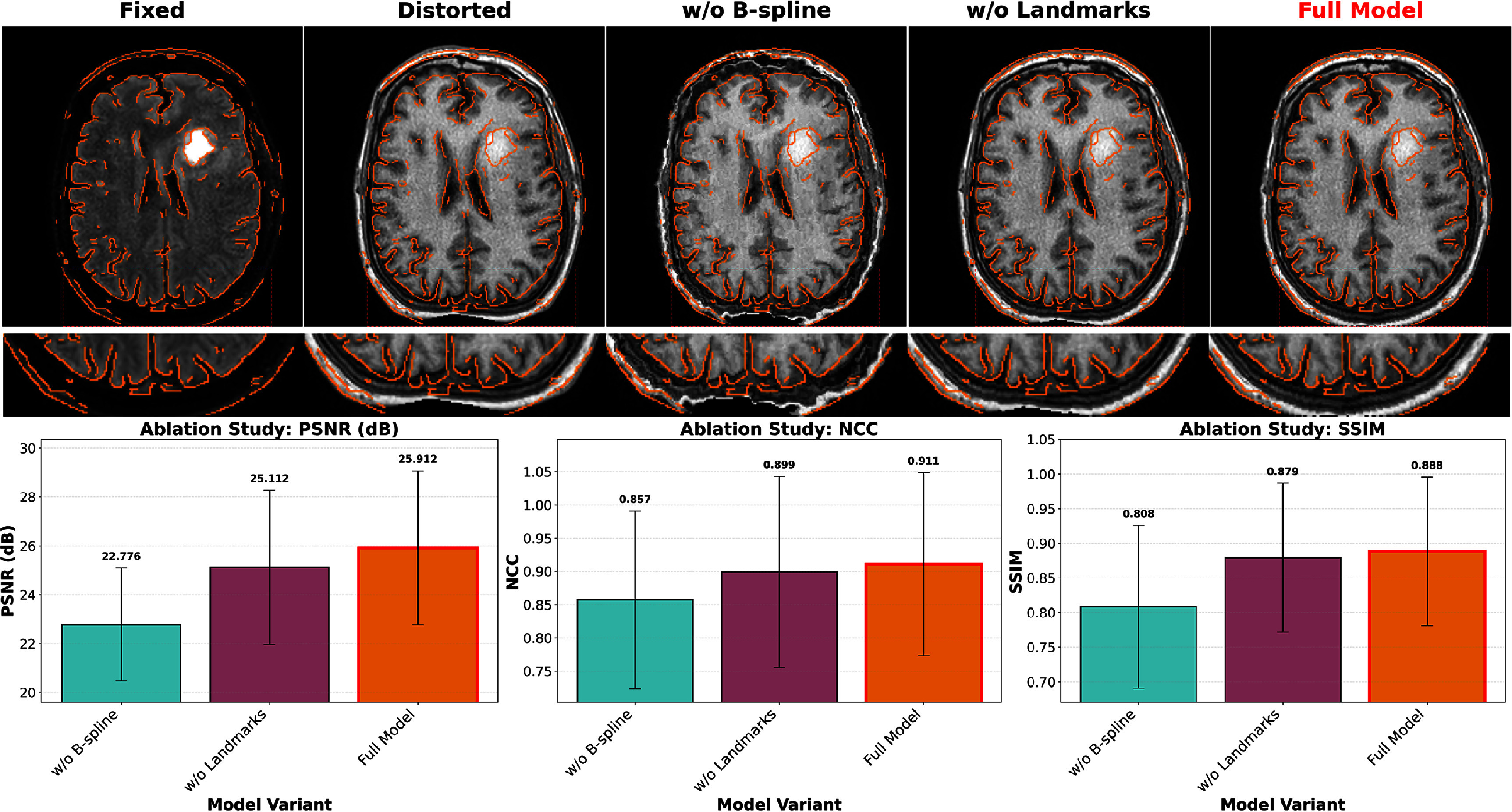
Ablation study demonstrating component contributions. Top row shows representative registration results for three configurations: full model, without B-spline regularization, and without landmark guidance. Bottom row presents quantitative metrics across the simulation dataset.

Systematic ablation experiments evaluated the individual contributions of landmark guidance and B-spline parameterization to overall registration performance. Figure 9 presents both quantitative metrics and representative visual results, demonstrating the necessity of each component. The full LMBS-INR model achieved an average PSNR of 25.912 dB, NCC of 0.911, and SSIM of 0.888. Removing B-spline regularization significantly degraded performance to PSNR of 22.776 dB, NCC of 0.857, and SSIM of 0.808. Eliminating landmark guidance resulted in intermediate performance with PSNR of 25.112 dB, NCC of 0.899, and SSIM of 0.879.

The visual results in figure 9 clearly illustrate the failure modes of incomplete models. Without B-spline regularization, the unconstrained neural network produces high-frequency artifacts and discontinuous deformations that violate physical constraints of SS-EPI distortions. The SSIM metric showed particular sensitivity to this degradation, dropping from 0.888 to 0.808. Conversely, removing landmark guidance showed a smaller performance drop (SSIM from 0.888 to 0.879), but leads to failures in regions where MI cannot establish correct correspondence. As shown in the magnified regions of the visualization, the outer brain regions in the fixed image appear very dark (approaching background intensity), while the corresponding skull regions in the distorted image exhibit high intensity. MI struggles to work correctly in such regions with complex intensity relationships. With the assistance of landmark matching models that possess higher-dimensional anatomical knowledge, our method achieves better performance in these challenging areas.

### 3.6. Parameter sensitivity analysis

#### 3.6.1. Landmark loss weight optimization

**Figure 10.**
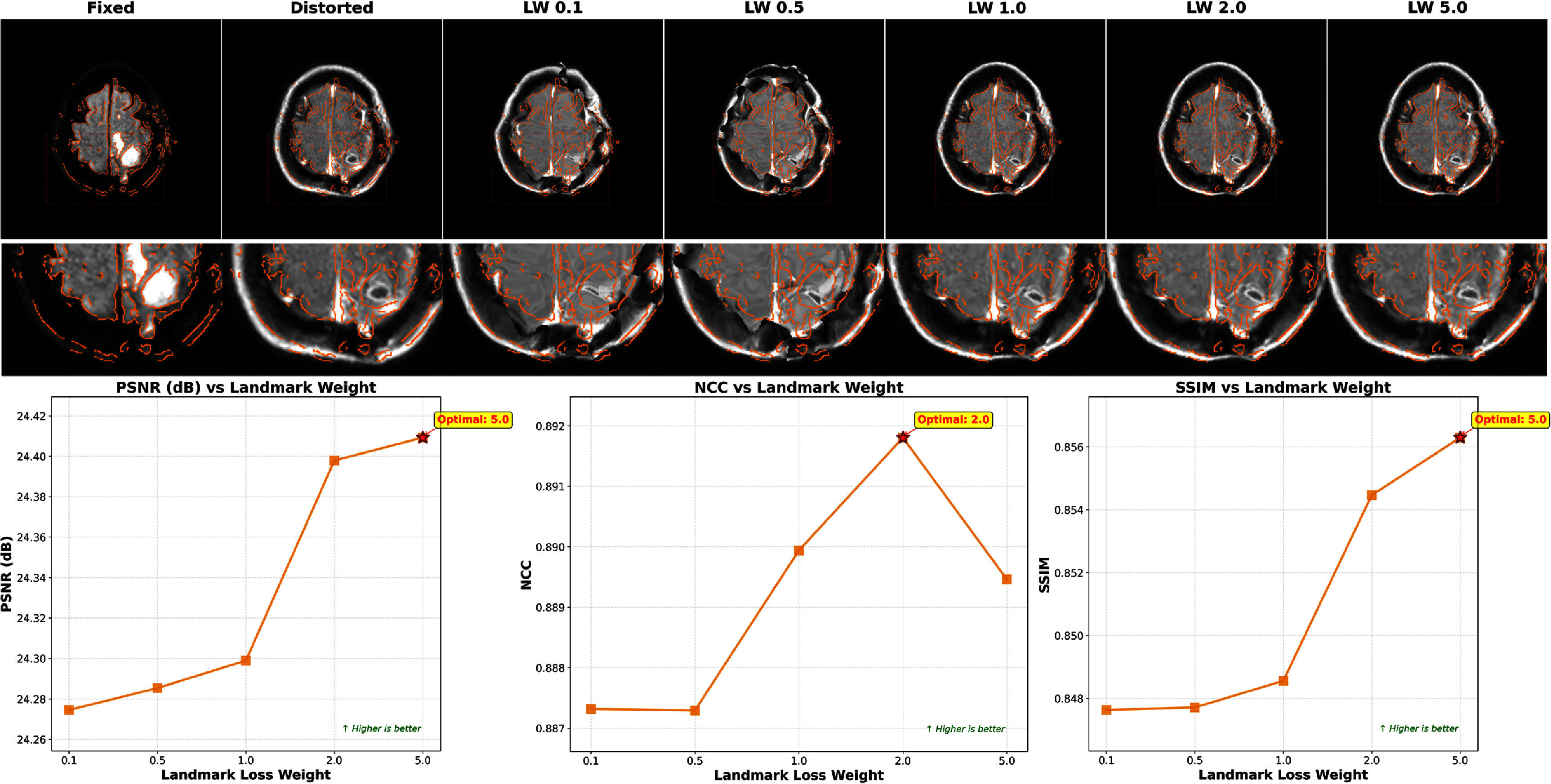
Impact of the landmark loss weight on registration accuracy. Performance metrics (PSNR, NCC, and SSIM) are evaluated across different $\lambda_\mathrm {LD}$ values. Visual results demonstrate that low weights lead to unrealistic local deformations, while increased weights help leverage foundation model knowledge to overcome mutual information limitations.

The landmark loss weight $\lambda_\mathrm {LD}$ represents a critical hyperparameter balancing anatomical correspondence constraints against intensity-based similarity. Figure 10 presents comprehensive evaluation across weights ranging from 0.1 to 5.0. While PSNR and SSIM metrics reached their peak at $\lambda_\mathrm {LD} = 5.0$, NCC achieved optimal performance at $\lambda_\mathrm {LD} = 2.0$. Considering the balance across all metrics and visual quality, we selected $\lambda_\mathrm {LD} = 2.0$ for all experiments.

Visual analysis revealed distinct failure modes at extreme values. With insufficient weighting ($\lambda_\mathrm {LD} < 0.5$), the model produced unrealistic local deformations, converging to local-minima characteristic of pure MI optimization. This limitation is particularly evident in skull regions where the fixed image shows dark skull areas similar to background, while the distorted image displays skull intensity similar to brain tissue-a challenge for MI-based registration. As landmark weight increases, the foundation model’s knowledge progressively helps overcome these MI limitations, correcting misalignments in ambiguous intensity regions. The selected value of $\lambda_\mathrm {LD} = 2.0$ achieved effective balance across all evaluation metrics.

#### 3.6.2. B-spline control point spacing

**Figure 11.**
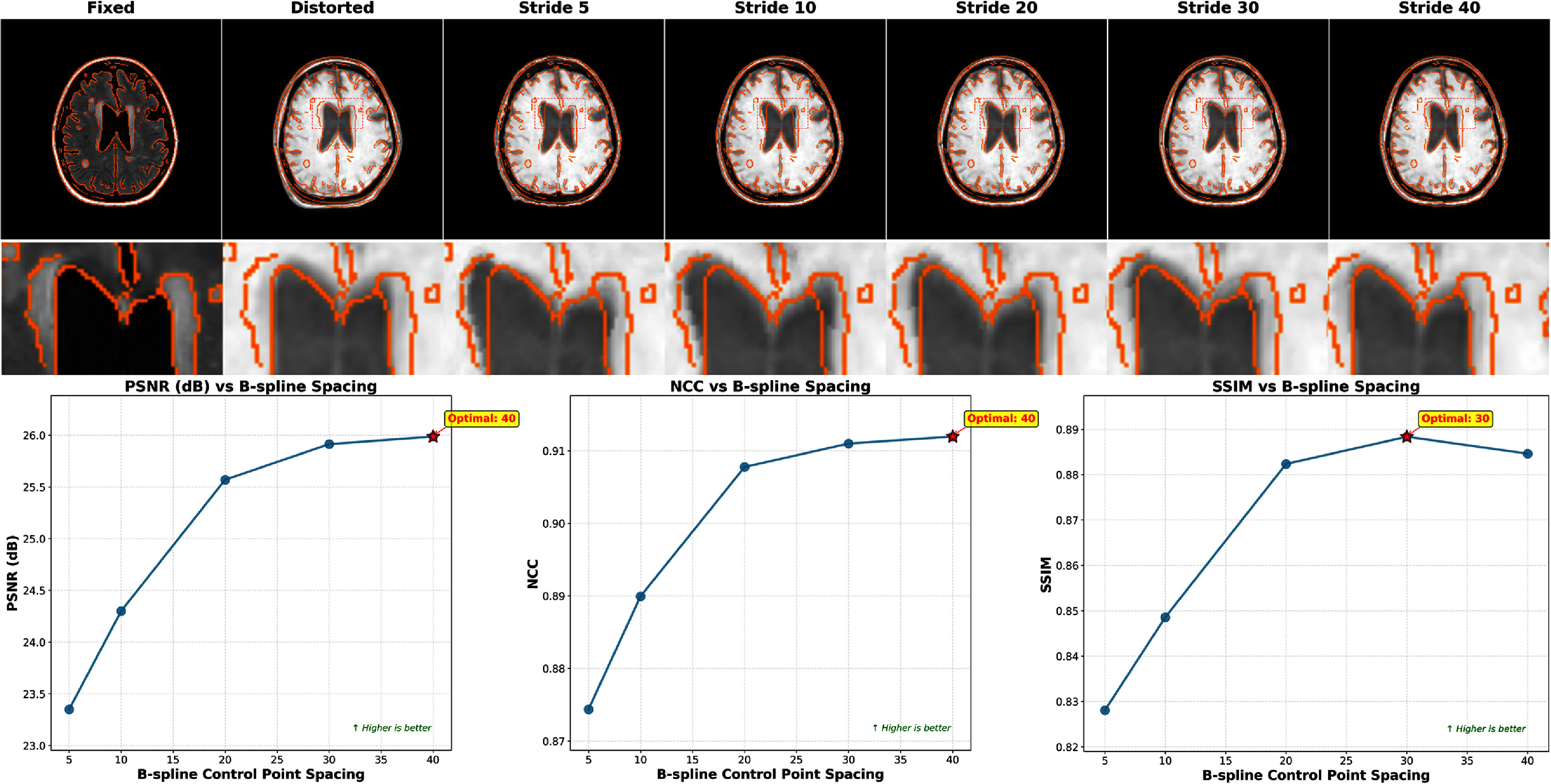
Effect of B-spline control point spacing on registration performance. Metrics including PSNR, NCC, and SSIM are evaluated across different spacing values.

Control point spacing fundamentally determines the deformation field’s flexibility and smoothness characteristics. Figure 11 illustrates performance variation across spacings from 5 to 40 voxels. The analysis revealed a clear trade-off between capturing fine-scale distortions and maintaining physically plausible deformations. Optimal performance emerged at 30 or 40-voxel spacing. Since larger spacings may fail to capture fine deformations and the performance difference between 30 and 40 voxels was minimal, we selected 30 voxels for all experiments in this study. Fine spacing (5-10 voxels) enabled modeling of high-frequency deformations but increased the susceptibility to overfitting, producing erroneous deformations. As shown in the visualization, spacings of 5 and 10 voxels resulted in incorrect registration of cerebrospinal fluid regions due to overfitting.

## 4. Discussion

The landmark matching B-spline INR (LMBS-INR) framework proposed in this study effectively addresses fundamental challenges in multimodal DWI distortion correction. By integrating cross-modal landmark correspondences with physically-constrained deformation modeling, our approach overcomes the inherent limitations of purely intensity-based registration methods. The consistent performance improvements across simulated and clinical datasets demonstrate the robustness and clinical applicability of this integrated approach. The primary technical innovation lies in the synergistic combination of foundation model features with traditional registration objectives. While MI has long served as the standard metric for multimodal registration, our results confirm its susceptibility to local minima and non-smooth solutions when used in isolation. The landmark guidance from pre-trained matchers provides crucial anatomical anchoring precisely where intensity relationships are ambiguous. This complementary information source proves particularly valuable in regions of low contrast, such as deep gray matter structures, where conventional methods frequently fail. Furthermore, the B-spline parameterization enforces physically plausible deformations consistent with the smooth spatial variations of magnetic field inhomogeneities, preventing the overfitting common in unconstrained optimization approaches.

Despite these significant advances, several limitations warrant discussion. First, a current implementation choice is the use of a fixed control-point spacing for the B-spline grid. This entails a trade-off: smaller spacing captures finer deformations but risks overfitting, whereas larger spacing enhances regularization but may miss local details. A natural extension is to adapt the spacing to image content or uncertainty, which we leave for future work. Second, our multi-view landmark extraction strategy relies on 2D feature matching across orthogonal slices rather than direct 3D correspondence estimation. Three-dimensional cross-modal feature matching remains an open challenge in computer vision and medical imaging, due to issues including significantly higher computational complexity, more intricate geometric transformations, and limited availability of diverse training data. Future developments in robust 3D cross-modal matchers could directly replace this component and potentially enhance registration accuracy. Third, while the current method performs patient-specific optimization very quickly, transfer learning strategies involving pre-training on multi-patient datasets followed by patient-specific fine-tuning could further improve efficiency. Fourth, our method primarily addresses susceptibility-induced geometric distortions (B0 field inhomogeneities) without considering eddy current-induced distortions, which may become significant at high *b*-values exceeding 1000 s mm^−2^, though this is beyond the scope of our radiotherapy clinical protocols.

## 5. Conclusion

This study presents a novel framework combining landmark-guided registration with B-spline parameterized INRs for robust DWI distortion correction. The method successfully addresses the fundamental challenge of multimodal registration between distorted DWI and undistorted anatomical references by leveraging complementary information sources: learned cross-modal correspondences provide global anatomical constraints, while B-spline regularization ensures locally smooth, physically plausible deformations. Comprehensive evaluation on simulated and clinical datasets demonstrated statistically significant improvements over state-of-the-art methods. The improved geometric accuracy achieved by our method has direct implications for radiation therapy planning, where precise tumor delineation and functional target mapping are essential for determining treatment margins and assessing therapeutic response.

## Data Availability

The data cannot be made publicly available upon publication because they contain sensitive personal information. The data that support the findings of this study are available upon reasonable request from the authors.
